# Primate Amygdalo-Nigral Pathway for Boosting Oculomotor Action in Motivating Situations

**DOI:** 10.1016/j.isci.2020.101194

**Published:** 2020-05-23

**Authors:** Kazutaka Maeda, Ken-ichi Inoue, Jun Kunimatsu, Masahiko Takada, Okihide Hikosaka

**Affiliations:** 1 Laboratory of Sensorimotor Research, National Eye Institute, National Institutes of Health, Bethesda, MD 20892, USA; 2 Systems Neuroscience Section, Primate Research Institute, Kyoto University, Inuyama, Aichi 484-8506, Japan; 3 PRESTO, Japan Science and Technology Agency, Kawaguchi, Saitama 332-0012, Japan; 4 Division of Biomedical Science, Faculty of Medicine, University of Tsukuba, Tsukuba, Ibaraki 305-8577, Japan; 5 Transborder Medical Research Center, University of Tsukuba, Tsukuba, Ibaraki 305-8577, Japan

**Keywords:** Neuroscience, Behavioral Neuroscience, Neuroanatomy

## Abstract

A primary function of the primate amygdala is to modulate behavior based on emotional cues. To study the underlying neural mechanism, we first inactivated the amygdala locally and temporarily by injecting a GABA agonist. Then, saccadic eye movements and gaze were suppressed only on the contralateral side. Next, we performed optogenetic activation after injecting a viral vector into the amygdala. Optical stimulation in the amygdala excited amygdala neurons, whereas optical stimulation of axon terminals in the substantia nigra pars reticulata inhibited nigra neurons. Optical stimulation in either structure facilitated saccades to the contralateral side. These data suggest that the amygdala controls saccades and gaze through the basal ganglia output to the superior colliculus. Importantly, this amygdala-derived circuit mediates emotional context information, whereas the internal basal ganglia circuit mediates object value information. This finding demonstrates a basic mechanism whereby basal ganglia output can be modulated by other areas conveying distinct information.

## Highlights

• Amygdala facilitates saccades and gaze to the contralateral side
• This is mediated by direct inhibitory connection to substantia nigra pars reticulata
• Motivational context is transmitted through this pathway to control saccade and gaze
• Amygdala thus controls goal-directed behavior using the basal ganglia circuit

## Graphical Abstract

**Figure:**
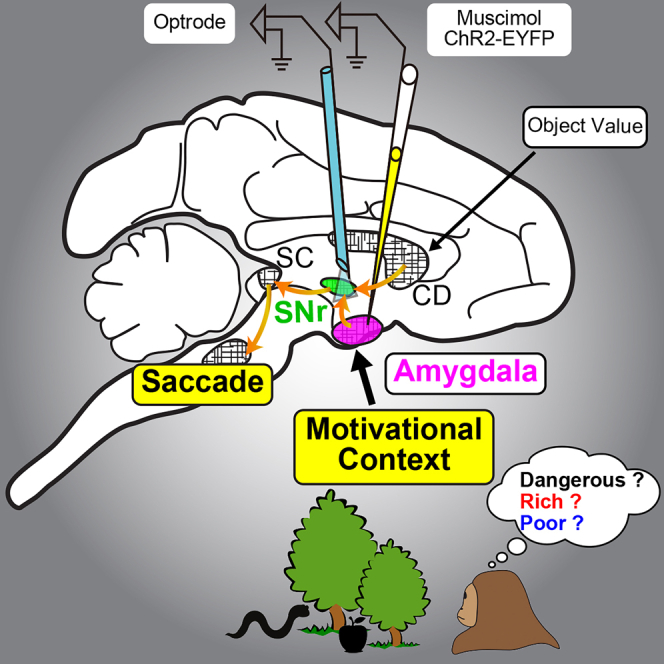

## Introduction

Eye movements are important for scanning the visual environment and making decisions. Abnormal eye movement patterns are a common symptom in many psychiatric disorders, including attention-deficit/hyperactivity disorder, bipolar disorder, and autism (Constantino et al., 2017, Munoz et al., 2003, Yep et al., 2018). Amygdala abnormalities are thought to be a key factor in these disorders (Avino et al., 2018, Davis and Whalen, 2001); however, it remains unknown whether the amygdala dysfunction and oculomotor symptoms are related to each other. Some studies have shown that amygdala lesions alter gaze patterns, especially for face images (Dal Monte et al., 2015, Taubert et al., 2018). Moreover, amygdala neurons are spatially selective and encode information about both the location and the motivational significance of visual cues (Peck and Salzman, 2014). As spatial attention is tightly coupled to motor function, especially in the case of stimulus-driven orienting behaviors (Corbetta and Shulman, 2002), it is plausible that amygdala neurons convey signals appropriate for control of eye movements. However, causal evidence linking the amygdala to abnormal eye movement patterns is lacking in both human and animal studies.

We have recently demonstrated that amygdala neurons, mostly in the central nucleus (CeA), encode emotional contexts (dangerous versus safe, rich versus poor) (Maeda et al., 2018). Importantly, we found that the activity of amygdala neurons was negatively correlated with the reaction time of saccadic eye movements to reward-associated objects. Anatomical studies have reported that the amygdala sends output to the basal ganglia, including the caudate tail (CDt), the globus pallidus externus (GPe), and the substantia nigra pars reticulata (SNr) (Fudge and Haber, 2000, Griggs et al., 2017, Price and Amaral, 1981, Shinonaga et al., 1992, Vankova et al., 1992). These structures are known to comprise a circuit that encodes the stable values of objects learned through long-term experience and also controls saccades through the SNr-superior colliculus (SC) pathway (Amita et al., 2019, Griggs et al., 2017). This suggests that amygdala neurons modulate saccadic eye movements by sending contextual information to the basal ganglia circuit.

Here we assessed the impact of motivating contexts on amygdala neuron activity through electrophysiological recordings and explored its relation to saccadic eye movements in a foraging task. Saccades to the contralateral side were strongly suppressed by muscimol-induced inactivation and enhanced by optogenetic activation of the amygdala. The results indicate that the amygdala controls the saccade and gaze position in a spatially selective manner. Furthermore, we provide evidence for the amygdala-SNr (amygdalo-nigral) pathway to control spatial attention and action in motivating contexts.

## Results

### Suppression of Saccades by Amygdala Inactivation

**Figure 1:**
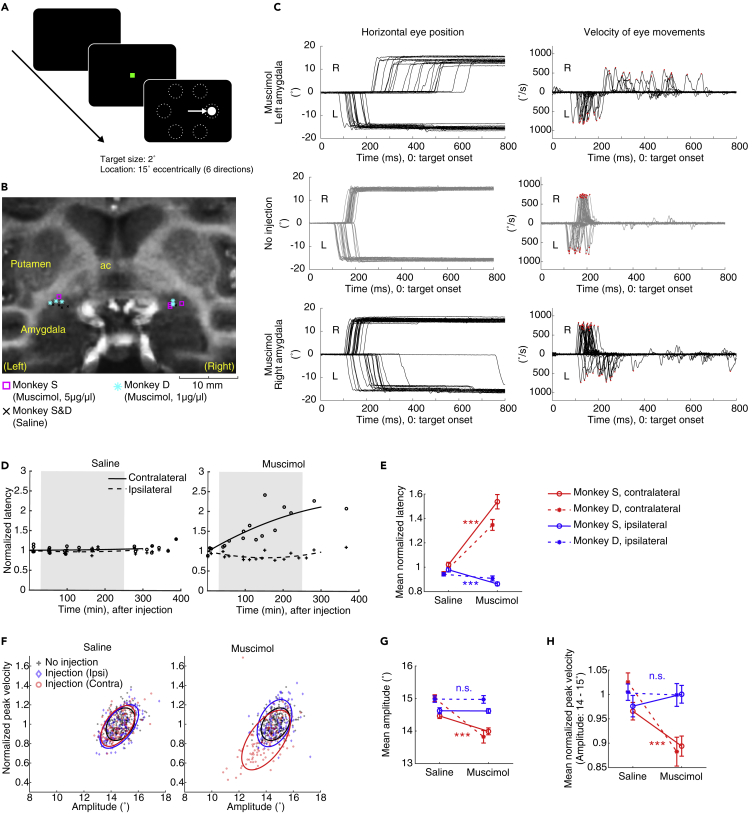
Changes in Saccade Features by Amygdala Inactivation (A) Visually guided saccade task. (B) Estimated injection sites in the central nucleus of amygdala. ac, anterior commissure. (C) Horizontal eye position (left) and velocity (right) after target onset (red dots: the peak velocity in each saccade). (D) Changes in saccade latency after injection of saline (left) and muscimol (right). Shaded gray area shows the effective period we used for further analysis (each data point: averaged saccade latency in each session). Solid and dashed lines indicate second-degree least-squares fit to the data points for contralateral and ipsilateral, respectively. (E) Mean latency in each condition. The data were obtained from individual trials during the effective period. (F) Relationship between saccade amplitude (abscissa) and peak velocity (ordinate). Solid lines show 70% confidence ellipse in each condition (black: no injection, red: CeA inactivation—contralateral saccades, blue: CeA inactivation—ipsilateral saccades). (G) Mean saccade amplitude. (H) Mean saccade peak velocity. Error bars show SEM. Asterisk (∗∗∗) indicates statistically significant contrasts at p < 0.001 (two-sample t test). All data in C, D, and F are for monkey S (Figure S1 show original data for both monkeys S and D).

We first examined the behavioral function of the amygdala by temporarily inactivating amygdala neurons through local injection of muscimol, a GABA_A_ receptor agonist (Hikosaka and Wurtz, 1985a, Hikosaka and Wurtz, 1985b). Before and after the muscimol injection (8.8 or 44 nmol [1μg or 5 μg] in a 1μL volume), monkeys performed a visually guided saccade task (Figure 1A). Before each injection, we identified the amygdala physiologically by recording neuronal activity using an injectrode (polyimide tube attached to a recording electrode for drug delivery, see Methods). We carried out 8 unilateral injections in monkey S (44 nmol [5μg] muscimol: 4 times, saline: 4 times) and 12 unilateral injections in monkey D (8.8 nmol [1 μg] muscimol: 6 times, saline: 6 times) (Table S1). Figure 1B shows the injection sites that appeared to be located in an amygdala sector corresponding to CeA in a magnetic resonance (MR) image.

In the visually guided saccade task, unilateral injections of muscimol into the amygdala suppressed contralateral saccades. Examples from a typical session are shown in Figure 1C: left amygdala inactivation (Figure 1C, top) increased the latencies of saccades to the right (contralateral) target compared with control data (Figure 1C, middle). Moreover, the saccade peak velocity decreased during such delayed saccades (Figure 1C, top right). In contrast, the saccade velocity to the left (ipsilateral) target was unchanged, although latencies were slightly shortened. Right amygdala inactivation (Figure 1C, bottom) produced similar effects: longer latencies and lower peak velocities for saccades to the left (contralateral) target and slightly shorter latencies for saccades to the right (ipsilateral) target.

We repeated the same experiment with varying delays after muscimol injections. The effect of muscimol on saccade latency started immediately and increased over time within a daily session (Figures 1D and S1A for original data). We analyzed the data in an effective period (30–250 min after the injection) and compared with the effect in the same window in control experiments (saline injection). The latency increased significantly on the contralateral side during amygdala inactivation, and the results were similar in both monkeys (Figure 1E, two-sample t test, T(397) = −11.385, p = 3.518 × 10^−26^, individual statistics values written below are also shown in Table S2). On the ipsilateral side, the latency tended to decrease during amygdala inactivation.

In addition to the latency changes described above, the amplitude of contralateral saccades sometimes became smaller (Figures 1F and 1G, two-sample t test, T(397) = 6.7242, p = 6.1672 × 10^−11^), i.e., hypometric following the muscimol injection (Figure 1C, top and bottom) compared with control data (Figure 1C, middle). We considered the possibility that the lower peak velocity of contralateral saccades might simply be correlated with smaller saccade amplitudes according to the main sequence (Pearson's correlation, r > 0, p < 0.05) (Figure 1F), a well-known property of saccades (Baloh et al., 1975, Robinson, 1964). To test this possibility, we compared the peak velocity within a short range of saccade amplitude (14–15°) and found that the peak velocity still significantly decreased following inactivation of the amygdala in both monkeys (Figure 1H, two-sample t test, T(112.8057) = 4.6518, p = 9.0142 × 10^−6^). These data indicate that inactivation of the amygdala alters three properties of contralateral saccades: (1) increase in latency; (2) decrease in amplitude; and (3) decrease in peak velocity.

Our data showed that inactivation of the amygdala suppressed oculomotor behaviors. However, this effect might be caused by the inactivation of surrounding areas, especially the caudal-ventral part of the putamen, which we call “putamen tail (PUTt)” where neurons encode stable object values (Kunimatsu et al., 2019). We thus injected muscimol in PUTt unilaterally (monkey S: n = 3, monkey D: n = 4) (Figure S2A). Although the saccade latency increased after muscimol injections in PUTt (nested data: p = 1.5389 × 10^−10^, monkey S: p = 3.8975 × 10^−7^, monkey D: p = 2.3778 × 10^−6^), the effects were weaker and shorter than after muscimol injections in amygdala (nested data: p = 9.8533 × 10^−5^, monkey S: p = 0.15483, monkey D: p = 1.558 × 10^−5^) (Figures S2B and S2C). We also injected muscimol in GPe (i.e., dorsal to amygdala), which again caused only weak effects on saccade latency (Figure S2B, right). We decided not to repeat this experiment because it caused long-lasting contralateral hemiplegia (around 2 weeks) with no further change in saccades, suggesting that this part of GPe controls body movements rather than eye movements. These data suggest that the full strength of the effects of muscimol on saccades must indeed be due to the amygdala inactivation.

The saccade deficits might be caused by the dysfunction of visual perception rather than the dysfunction of oculomotor control. To test this possibility, we used the value-based visually guided saccade task (Figure S3A). Two dots in different colors were used as the good and bad objects, which were associated with large and small rewards. Normally (with no or saline injection), the monkeys made saccades quickly (short latency) to the good object and slowly (long latency) to the bad object (Figure S3B, left), as shown previously (Kim and Hikosaka, 2013, Yamamoto et al., 2013). After muscimol injection into the amygdala, the saccade latency increased for both objects on the contralateral side. However, it remained shorter for the good object than for the bad object on both the ipsilateral and the contralateral sides (Figure S3B, right). The data suggest that the visual perception of the target in the visually guided saccade task (Figure 1A) was kept intact even after the amygdala inactivation.

**Figure 2:**
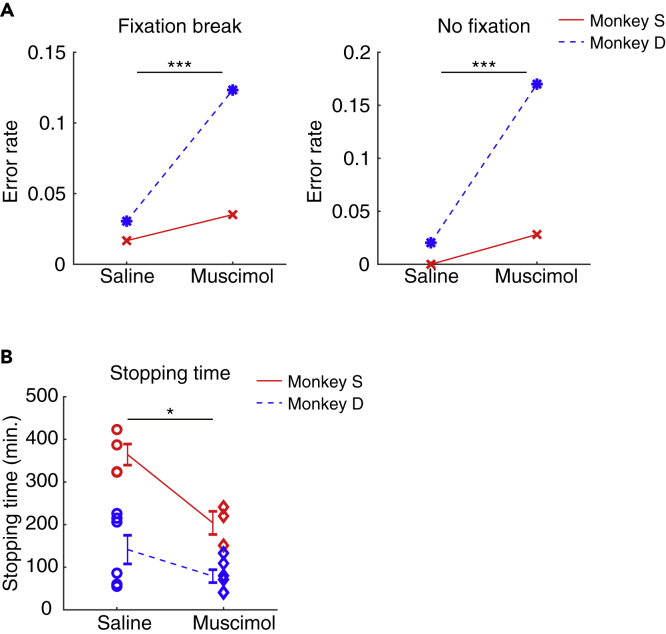
Motivational Deficits during Goal-Directed Behavior Following Amygdala Inactivation (A) Increase in fixation error rate before saccade target appeared: fixation breaks (left) and no fixation trials (right). (B) Earlier termination of the visually guided saccade task following amygdala inactivation. The stopping time was defined as the point at which the monkey failed to make the initial fixation on five consecutive trials over the course of an injection session. When no such sequence of failures occurred, the end of planned experimental session was taken as the stopping time (monkey S: 364.25 ± 49.30 min, monkey D: 131.29 ± 79.74 min in the control session). Each marker indicates the stopping time in each injection experiment (red: monkey S, blue: monkey D). Error bars show SEM; asterisks (∗) and (∗∗∗) indicate statistically significant contrasts at p < 0.05 and p < 0.001. related to Figure 1.

Inactivation of the amygdala also disrupted the monkeys' goal-directed visual behavior in ways not captured by the saccade metrics described above. On each trial of the visually guided saccade task (Figure 1A), the monkey needed to wait for the reward-associated object by maintaining gaze fixation on a central dot (770 ms), otherwise the trial ended with no opportunity for reward. Such adequate fixation was disrupted by amygdala inactivation in both monkeys (Figure 2A), in that they often broke fixation on the central dot before the object appeared (fixation break, nested data: p = 6.8931 × 10^−5^, monkey S: p = 0.13588, monkey D: p = 2.2485 × 10^−5^) or failed to look at the fixation point altogether (no fixation, nested data: p = 4.9368 × 10^−10^, monkey S: p = 0.0034666, monkey D: p = 5.5859 × 10^−10^). These effects, together with the preceding results (Figure 1), suggest that the amygdala contributes to the goal-directed behavior both by suppressing inappropriate saccades in the absence of valid targets and by facilitating appropriate saccades after targets appear.

Under normal conditions, the monkeys were sufficiently motivated to perform behavioral tasks continually for long durations. After muscimol injection into the amygdala, however, the monkeys were more prone to quit performing the tasks we tested, including the visually guided saccade task (Figure 2B, mean stopping time (saline versus muscimol): 230.5 min versus 120.7 min after the injection, nested data: p = 0.04047, monkey S: p = 0.0088632, monkey D: p = 0.13501). This result suggests that the amygdala may play a role in motivational activation of goal-directed behavior. We consider this possibility in the next section.

### Gaze Shift during Amygdala Inactivation

**Figure 3:**
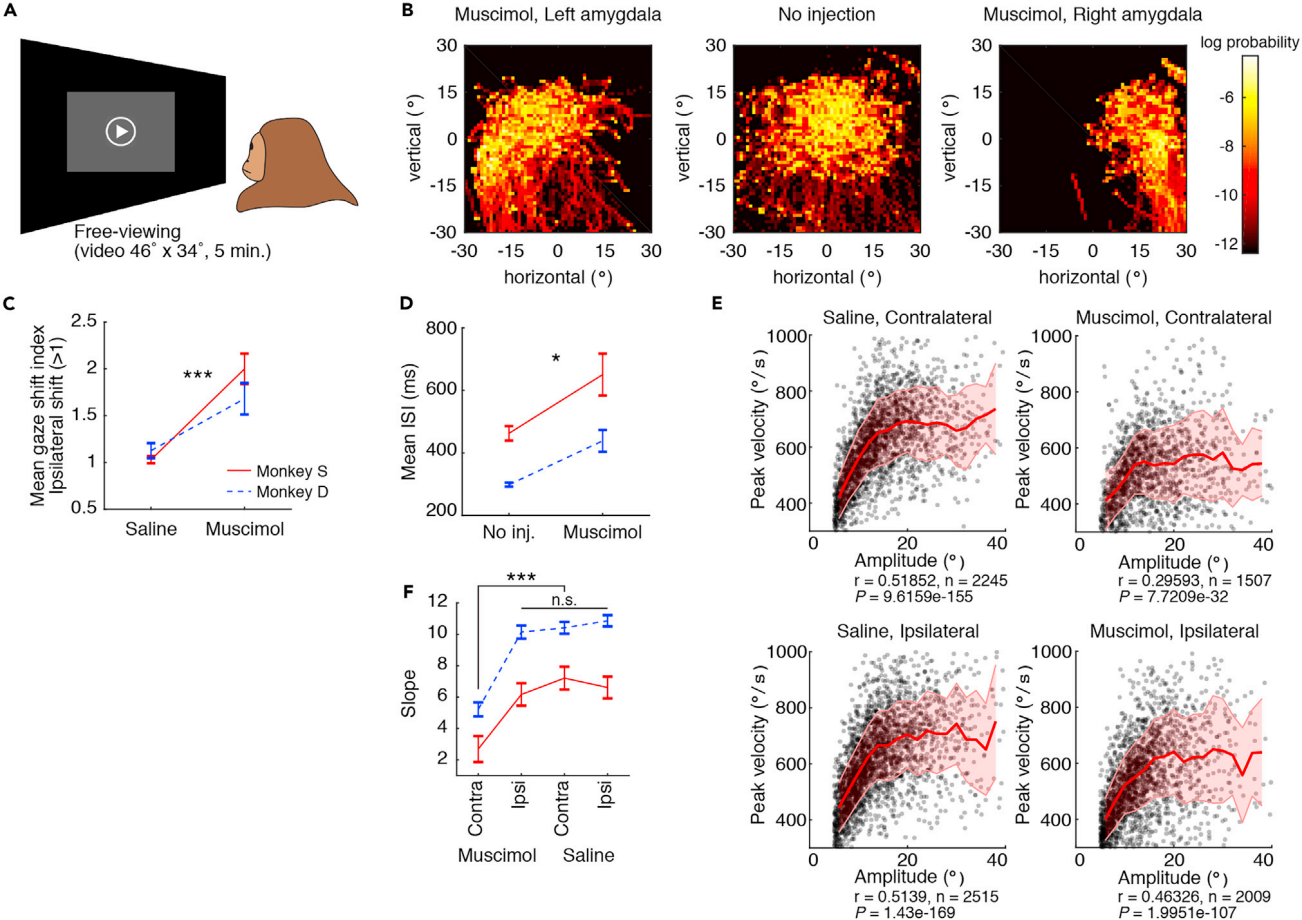
Changes in Free Viewing by Amygdala Inactivation (A) Free-viewing task. (B) Gaze positions during a video clip (5 min) in three conditions. The distribution probabilities over time after stimulus onset were calculated by logarithmic two-dimensional histograms of vertical and horizontal gaze directions. (C) Gaze shifts after saline or muscimol injection in the amygdala. The shift index was calculated in each session (see Methods) and the average is plotted. (D) Inter-saccade interval (ISI) after no injection or muscimol injection in the amygdala. (E) Relationship between saccade amplitude (abscissa) and peak velocity (ordinate). Solid lines show the average of peak velocity in each 2-degree bin of saccade amplitude. Shading shows SD (Pearson's correlation). (F) Slopes of regression lines (ANCOVA and post hoc: least significant difference) for the four groups shown in (E). Error bars in (C), (D), and (F) show SEM. Asterisks (∗) and (∗∗∗) indicate statistically significant contrasts at p < 0.05 and p < 0.001. All data in B and E are for monkey D (Figure S4 for monkey S).

Under natural viewing conditions, eye movements are used to look at things sequentially according to the animal's attentional and motivational states as well as object saliency (Corbetta et al., 1998, Hoffman and Subramaniam, 1995, Taubert et al., 2018). To test whether the amygdala contributes to such behavior, we let monkeys watch a video clip freely without any reward (free-viewing task, duration: 5 min, see Methods and Figure 3A). Each image in Figure 3B shows eye positions of one monkey (S) during the 5-min free-viewing task. The duration of gaze (i.e., fixation between saccades) is indicated by color map. Without inactivation (Figure 3B center), monkey's gaze was distributed in various positions but was more common around the center. In contrast, during left or right amygdala inactivation (Figure 3B, left or right), gaze was mostly on the left (ipsilateral) or right (ipsilateral) side, respectively. The bias toward gaze shifts to the ipsilateral side was significant in both monkeys (Figures 3B and S4A, also 3C). These results may be characterized as “contralateral hemineglect” (Behrmann et al., 1997, Miyashita et al., 1995).

Amygdala inactivation also had general effects on saccadic eye movements (Figures 3D–3F). First, the frequency of saccades decreased, as reflected by an increased inter-saccade interval (ISI) (Figure 3D). These results indicate that saccadic eye movements were generally suppressed, whereas gaze tended to stay on the ipsilateral side. This effect may be related to attention and motivation (see Discussion). Second, saccades became slower. Because the saccade amplitude varied during free viewing, the relationship between the saccade velocity and the amplitude was well estimated in each of four conditions (Figure 3E). The peak velocity was positively correlated with the amplitude in all conditions (Pearson's correlation, r > 0, p < 0.001). However, the slope of the regression line decreased significantly only in contralateral saccades during amygdala inactivation (Figure 3F, ANCOVA and post hoc: least significant difference). These results indicate that the saccade peak velocity decreased across different amplitudes only in saccades directed to the side contralateral to the amygdala inactivation, which also confirmed the conclusion based on explicitly prompted saccades to targets in the visually guided saccade task (Figure 1).

### Optogenetic Activation of Amygdala

**Figure 4:**
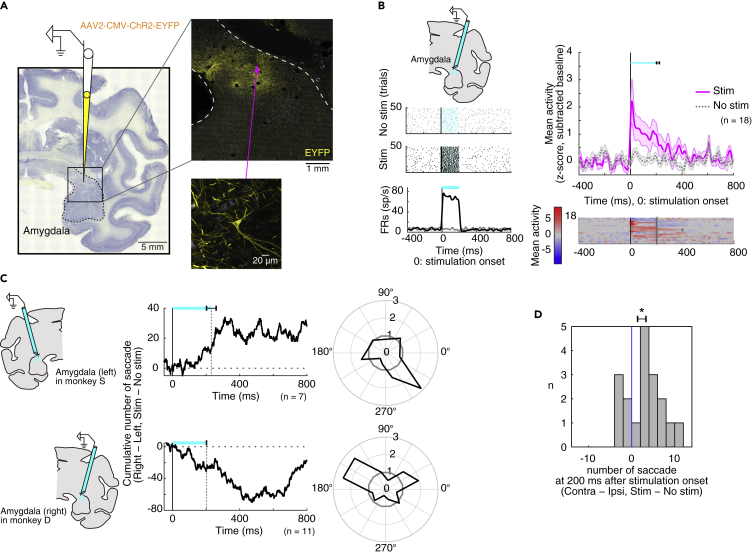
Optogenetic Activation of Amygdala Facilitates Contralateral Saccades (A) Injection site (left) and AAV2-CMV-ChR2-EYFP infections (right) in the amygdala (yellow: EYFP, blue: autofluorescence). Right-bottom shows EYFP expressions in a cell body of a CeA neuron. (B) Effects of optical stimulation on amygdala neurons. Left: activity of a CeA neuron around the no-stimulation period (top) and the stimulation period (200 ms) (center). Changes in firing rate in the stimulation/no-stimulation conditions are shown at bottom. Right: activity of 18 amygdala neurons. Population activity is shown at top. Spike activity was smoothed with a Gaussian kernel (σ = 10 ms). The cyan bar shows mean stimulation durations, and the error bar shows SEM. Responses of individual neurons (bottom) were converted to color scale and sorted by modulation latencies. Dots indicate the end of optical stimulation. The duration of optical stimulation was about 200 ms, except for one neuron (400 ms). (C) Directional bias of saccades by amygdala optical stimulation. In monkey S (upper), stimulation was in the left amygdala and saccades were biased to the right. In monkey D (lower), stimulation was in the right amygdala and saccades were biased to the left. Center: cumulative direction bias, which is aligned on the onset of optical stimulation. Right: polar plot showing ratio of saccades in radial directions in stimulation(+) versus stimulation(−) conditions (bin width = 30 deg). Values greater than 1 indicate more saccades in the stimulation(+) condition. (D) Bias of the number of saccades during the stimulation to each amygdala neuron (right-tailed one-sample t test). The error bar shows SEM. Asterisk (∗) indicates statistically significant contrasts at p < 0.05.

To further investigate the role of the amygdala in eye movement control and shed light on the neural pathways involved, we injected an adeno-associated virus type 2 vector (AAV2-CMV-ChR2-EYFP) into the amygdala of one hemisphere in both monkeys (monkey S: left, monkey D: right, Table S3). Figure 4A shows the expression of ChR2-EYFP in monkey D. Many ChR2-positive neurons were found in the amygdala, corresponding to its central nucleus (CeA), which was similar to the muscimol injection sites (Figure 1B). In order to gauge the efficiency of viral labeling, we counted the fraction of NeuN-positive cells that expressed ChR2-EYFP (example cells are shown in Figure S5A). Near centers of injection sites (0.5 mm × 0.5 mm), 55.7% (59/106) of the NeuN-positive cells expressed ChR2-EYFP. Thus, the viral vector enabled the expression of ChR2-EYFP in amygdala neurons of the monkey.

One month after the injections, we tested the effect of stimulation at the viral vector injection sites using optrodes to deliver 473-nm blue laser light while simultaneously recording single neuron activity in the amygdala, mostly in CeA (monkey S, n = 7; monkey D, n = 11, Figure S5B). Many of the neurons (13 of 18, monkey S, n = 7; monkey D, n = 6) were modulated by the stimulation, mostly with excitatory responses (11 of 13, monkey S: 5, monkey D: 6), indicating that the excitatory opsin was successfully expressed in this area. A representative neuron is shown in Figure 4B (left), which was excited tonically during the stimulation (t test, T(98) = −58.1173, p = 9.3491 × 10^−78^). The population activity of 18 neurons recorded is shown in Figure 4B (right).

Moreover, the optical stimulation of the amygdala induced contralateral saccades (Figure 4C) while the monkey was freely viewing. Both monkeys made saccades more frequently to the side contralateral to the stimulation (Figure 4C upper, monkey S; Figure 4C lower, monkey D). This effect started soon after the onset of 200-ms optical stimulation pulses and persisted for at least 400 ms. The contralateral facilitation commonly appeared across sessions and monkeys (Figure 4D, right-tailed one-sample t test, nested data: p = 0.014391, monkey S: p = 0.18396, monkey D: p = 0.019764). These results were complementary to the effect of the muscimol injection, indicating that the amygdala facilitates saccadic eye movements to the contralateral side.

As a control, we assessed the effects of optical stimulation without viral vector expression on the contralateral side in monkey S and in another monkey. Example neurons and saccadic biases are shown in Figure S6, which did not exhibit any clear modulation. Thus, the laser light by itself did not affect either neuronal activity or animal's behavior.

### Activation of Amygdalo-Nigral Pathway

Having established the efficacy of our viral vector injection, we proceeded to ask what specific circuit is implicated in facilitating contralateral saccades. It has been shown anatomically that CeA projects to SNr (Fudge and Haber, 2000, Gerfen et al., 1982, Price and Amaral, 1981, Shinonaga et al., 1992), which in turn controls saccades through its inhibitory connection to SC (Hikosaka et al., 2000, Hikosaka and Wurtz, 1983). To manipulate this circuit, we used optrodes to test how SNr neurons responded to optical stimulation of axon terminals that originated from the amygdala and expressed ChR2.

**Figure 5:**
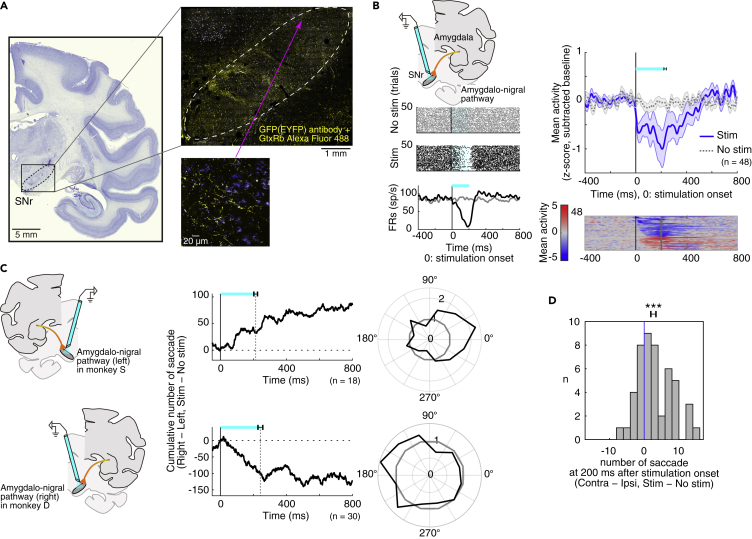
Amygdalo-Nigral Inhibitory Pathway Modulates Oculomotor Behavior (A) Enhanced anterograde transport of AAV2-CMV-ChR2-EYFP infection in SNr (yellow: GFP(EYFP) antibody + GtxRb Alexa Fluor 488, blue: autofluorescence). Right-bottom shows the expressions in axons. (B–D) SNr neuronal modulation and directional bias of saccades after optical stimulation of the amygdalo-nigral pathway. Same format as in Figures 4B–4D. (B, right) Activity of 48 SNr neurons that were excited or inhibited by optogenetic stimulation (inhibition: n = 34, excitation: n = 14). (D) Error bars show SEM. Asterisks (∗∗∗) indicate statistically significant contrasts at p < 0.001.

We examined neurons in the wide area of SNr (monkey S, n = 46; monkey D, n = 46), and found that about half of the SNr neurons were affected by optical stimulation (48/92, monkey S: 18, monkey D: 30), suggesting that they received inputs from the amygdala. Figure 5B (left) shows the response of a typical SNr neuron, which was inhibited during the optical stimulation. Most neurons tested were inhibited by the stimulation (n = 34/48, monkey S: 15, monkey D: 19), although some neurons were excited (Figure 5B, right-lower, n = 14/48, monkey S: 3, monkey D: 11). Both groups of neurons were distributed widely in SNr (Figure S5C). On average the population of SNr neurons were inhibited during the optical stimulation, which diminished gradually after the offset of the light pulse (Figure 5B, right-upper). These results suggest that a number of neurons in CeA have direct connections to SNr neurons, predominantly with inhibitory synapses. Indeed, many ChR2-positive axon terminals were found in SNr (Figure 5A).

We found that optical stimulation in SNr induced contralateral saccades in both monkeys (Figure 5C). This contralateral facilitation was significant across sessions (Figure 5D, right-tailed one-sample t test, nested data: p = 0.0003963, monkey S: p = 0.0074748, monkey D: p = 0.0067764). The results were similar to those obtained with the optical stimulation in the amygdala (Figures 4C and 4D). These data together suggest that the amygdala, especially CeA, facilitates contralateral saccades through its inhibitory connection to SNr. According to this model, saccades would be permitted by a disinhibition of SC neurons (Figure 7).

### Amygdalo-Nigral Activity in Motivating Contexts

**Figure 6:**
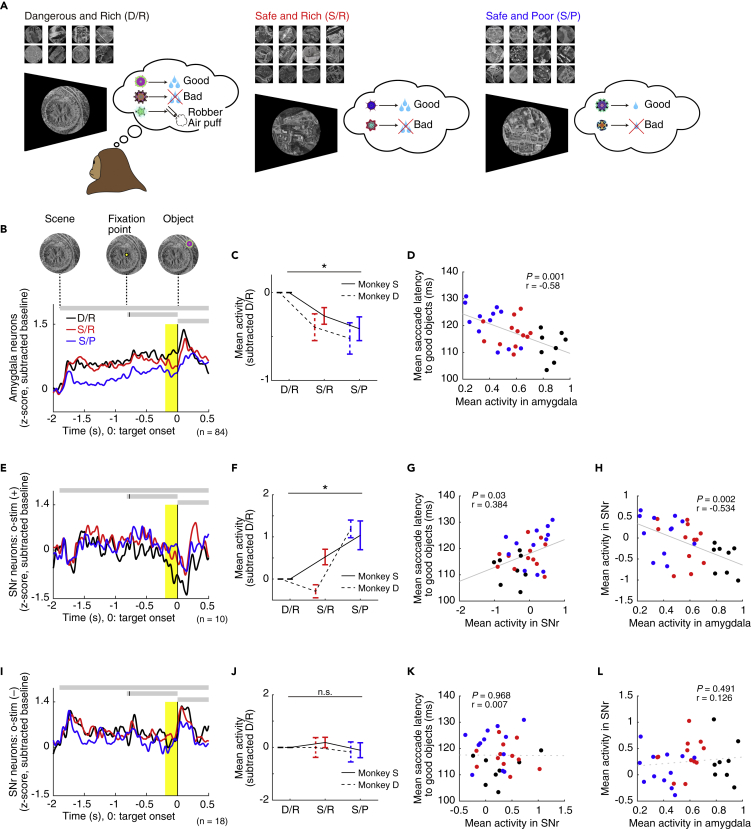
Amygdala Is Excited and SNr Is Inhibited by Motivating Contexts (A) Foraging task in three different contexts (D/R: 8, S/R: 12, S/P: 12 scenes). (B–D) Population activity of amygdala neurons (black: D/R, red: S/R, blue: S/P). (B) Activity changes of amygdala neurons during the period including the onsets of scene, fixation point, and object in monkey S. (C) Mean amygdala activity in each context. This was calculated during the period 200 ms before the object onset (shaded yellow area in B). Error bars indicate SEM. (D) Relation between the mean amygdala activity (abscissa) and mean saccade latency (ordinate) (Pearson's correlation) in monkey S. Data are presented separately for individual scenes that are shown in (A). (E–H) Population activity of SNr neurons that were optogenetically manipulated. E–G, same format as in B–D. (H) Relation between the mean amygdala activity (abscissa) and mean SNr activity (ordinate) for individual scenes (Pearson's correlation). (I–L) Population activity of SNr neurons that were not optogenetically manipulated. I–K, same format as in B–D. (L) Relation between the mean amygdala activity (abscissa) and mean SNr activity (ordinate) for individual scenes (Pearson's correlation). All neuronal data are for monkey S (Figure S7 for monkey D).

An important question arises as to what type of information is sent from the amygdala to SNr. Data in Figure S3 suggested that the amygdala, especially CeA, does not modulate saccades based on object values, unlike the basal ganglia (Kim and Hikosaka, 2013, Tachibana and Hikosaka, 2012). Using a scene-based foraging task, we previously found that amygdala neurons tended to be activated in motivating contexts (Maeda et al., 2018). Here, we examined activity of amygdala neurons as well as SNr neurons using the same foraging task (Figure 6A).

In each trial of the task, the screen in front of the monkey was dark at first. Next, a large visual scene (chosen from satellite imagery) appeared suddenly, which acted as an environmental context. Each scene contained at least two fractal objects, which appeared one at a time, randomized in both sequence and position. Target objects were associated with either reward (water) or no reward, referred to as “good” and “bad” objects, respectively. A different fractal object called the "robber" could appear in some scenes, which signaled the threat of an air puff and possible cancellation of the reward. The monkeys were rewarded for making saccades to the good objects and holding fixation for 400 ms. The task had three different emotional contexts depending on the objects consistently associated with the scenes (Figure 6A): (1) D/R: dangerous (robber+) and rich (large reward), (2) S/R: safe (robber−) and rich (large reward), and (3) S/P: safe (robber−) and poor (small reward).

Amygdala neurons were most strongly activated with the D/R context, followed by the S/R and then the S/P context (Figures 6B, 6C, and S7C). Across all scenes tested, stronger neuronal responses were associated with shorter saccade latencies (Figures 6D and S7D). At the same time, neuronal activity was positively correlated with mean heart rate in each scene (Figure S7A), consistent with the expectation that amygdala activation was linked to emotional arousal. These data suggest that the emotional (i.e., motivating) contexts activate amygdala neurons, which then initiates saccades earlier.

The results from our optogenetic activation of axon terminals showed that the inhibitory connection from the amygdala to the SNr facilitated contralateral saccades (Figure 5). We therefore asked whether this neural pathway accounts for the modulation of saccades in emotional contexts. To address this question, we tested two groups of SNr neurons using the scene-based foraging task: (1) O-stim (+) neurons (i.e., activity affected by optogenetic stimulation; n = 17, monkey S: 10, monkey D: 7) (Figures 6E–6H and S7E–S7G); (2) O-stim (−) neurons (i.e., unaffected by optogenetic stimulation; n = 25, monkey S: 18, monkey D: 7) (Figures 6I–6L and S7H–S7J).

O-stim (+) SNr neurons, which were likely to receive direct inputs from the amygdala, were more strongly inhibited with the D/R or S/R context than with the S/P context in the absence of optical stimulation (Figures 6E, 6F, S7E). Moreover, lower activity of these neurons was associated with shorter saccadic latencies (Figure 6G and S7F). Finally, the activity changes across many scenes were negatively correlated between amygdala neurons and O-stim (+) SNr neurons (Figure 6H and S7G). In contrast, O-stim (−) SNr neurons, which were less likely to receive direct inputs from the amygdala, showed none of these effects: not modulated by contextual information (Figures 6I, 6J, and S7H) and not related to the saccade latencies and amygdala activities (Figures 6K, 6L, S7, and S7J).

**Figure 7:**
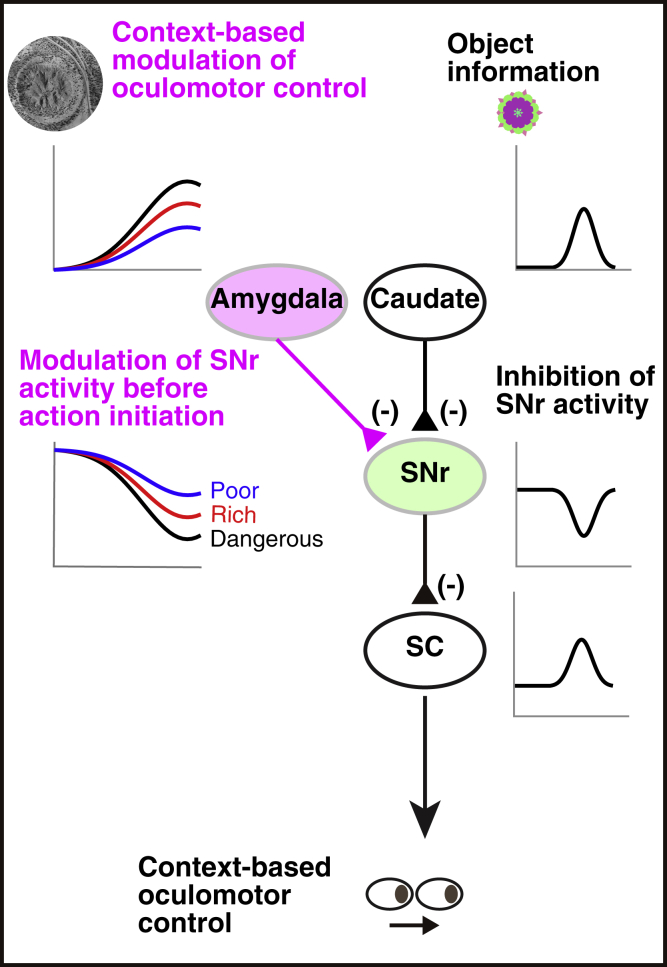
Hypothetical Neural Circuit SNr, substantia nigra pars reticulata; SC, superior colliculus.

These results reveal a neural circuit mechanism (amygdala-SNr-SC) that boosts the choice of good objects (by saccades) based on the motivational contexts (i.e., dangerous and rich) (Figure 7). Importantly, this mechanism is spatially selective (i.e., contralateral saccades), as required for the choice of one good object among competing alternative objects.

## Discussion

Our study defines a possible role of the amygdala in spatially selective oculomotor control based on contextual information. Central to this process is an amygdalo-nigral pathway that inhibits activity of SNr neurons (see Figure 7), contributing to disinhibition of SC neurons to generate saccades (Hikosaka and Wurtz, 1983). Consistent with this result, our pharmacological inactivation data showed that the amygdala inactivation suppressed saccades physically and motivationally (see Figures 1 and 2). Previous studies have revealed that neurons in the striatum (caudate nucleus and putamen) respond to many visual objects phasically and also encode the historical values of objects (Kim and Hikosaka, 2013, Kunimatsu et al., 2019, Yamamoto et al., 2013). The striatal neurons that have GABAergic (γ-aminobutyric-acid-releasing) projections to SNr control oculomotor behaviors based on the object information (Hikosaka et al., 2000, Yasuda et al., 2012). In the current findings, it is important to note that tonic activity of SNr neurons is modulated by emotional and motivational contexts (i.e., dangerous versus safe, rich versus poor) before object appearance and that the SNr neurons receive input from CeA encoding contextual information. These findings suggest that two different aspects of oculomotor control, pertaining to context and object, are mediated by distinct inhibitory inputs to SNr from the amygdala and striatum, respectively (see Figure 7).

Together with our previous findings (Maeda et al., 2018, Maeda et al., 2019), these results suggest that the amygdala governs social and emotional behaviors at multiple levels. At the most general level, the amygdala mediates context-appropriate social orienting, as evidenced by the effect of amygdala inactivation during movie free viewing (Maeda et al., 2019). At a more fine-grained level, the amygdala selectively mediates the spatially oriented action required for finding objects of interest quickly and accurately. This goal-directed aspect of amygdala function was disrupted by amygdala inactivation and facilitated by optogenetic activation of the amygdala or the amygdalo-nigral pathway. Both levels must work in tandem for appropriate behavior to take place, as the general promotion of a context-appropriate behavioral regimen must be followed by narrowly targeted actions aimed at choosing or rejecting particular objects. To this end behavior must be controlled accurately, especially in the spatial domain, otherwise random involuntary movements would prevail. For this reason, the spatially selective (i.e., contralateral) control of saccades by the amygdala is critical.

These data are relevant to the neural circuit concept proposed by Swanson and Petrovich (Swanson and Petrovich, 1998) that CeA in the rat is a region of the striatum specialized to modulate the autonomic motor outflow by inhibitory signals. In the monkey, CeA modulates goal-directed action (i.e., saccade and gaze) (see Figures 1, 2, 3, 4, and 5), possibly in addition to autonomic actions including the heart rate (see Figure S7A) and pupil size (see Figure 7 in a previous article (Maeda et al., 2018)). A recent study on the mouse revealed that CeA preferentially inhibited GABAergic neurons in the lateral substantia nigra, which corresponds to the lateral part of SNr in the monkey (Francois et al., 1984), and the circuit modulated emotional responses to salient stimuli (Steinberg et al., 2020). Such a coordination of autonomic and voluntary motor actions is crucial for responding appropriately to a given emotional/motivational context, which is utilized commonly in different animals.

These data suggest that the basal ganglia output can be modulated by other brain areas for goal-directed behavior. The final stage of the basal ganglia circuit, namely the SNr-SC pathway, has connection patterns well suited for promoting appropriate choices (Hikosaka et al., 2000): widespread inhibition of many unnecessary objects/actions and selective disinhibition of necessary objects/actions. Accordingly, brain areas outside the basal ganglia could participate in goal-directed behavior by sending inhibitory signals to SNr neurons, as the amygdala (especially CeA) does. Importantly, this same mechanism can be activated by different sources of information (e.g., object by striatum versus context by CeA). Our data, however, do not exclude the possibility that CeA may also use other targets (e.g., brainstem nuclei (Price and Amaral, 1981)) to control behaviors based on contexts.

A growing body of evidence suggests that the emotional or motivational information in the amygdala affects basal ganglia circuitry, and this modulation is critical for adjusting spatially selective oculomotor behaviors or attention in particular contextual environments. Dysregulation of the amygdala-basal ganglia linkage may contribute to psychiatric conditions, among which adjustment disorders in stressful environments are the most prevalent (Bachem and Casey, 2018, Zelviene and Kazlauskas, 2018). Interestingly, a recent study has shown that bilateral sensory stimulation reduces traumatic memory and facilitates adaptation to stressful environments by modulating a multisynaptic SC-mediodorsal thalamus-amygdala pathway (Baek et al., 2019). In humans, treatment regimens have been developed to utilize eye movements against stress disorders (i.e., eye movement desensitization and reprocessing) (Shapiro, 2001). Our present study defines that the amygdalo-nigral pathway in primates controls saccadic eye movements in stressful environments (or, motivating contexts) and that its dysfunction leads to behavioral impairments. A more precise functional understanding of this pathway will provide new insights into the possible mechanism underlying human psychiatric symptoms associated with adjustment disorders.

### Limitations of the Study

• This study shows the inhibitory effects of the amygdala on SNr neurons, which facilitates saccades to the contralateral side. However, there might be other effects of the amygdala on the basal ganglia that may be mediated by the connections to the striatum or GPe.
• We focused on the central nucleus of the amygdala but have not examined the amygdala as a whole. This could be tested by injecting viral vector in other amygdala nuclei.
• The optogenetic stimulation was performed with the single-unit electrophysiological recording, which may overlook the effect on neuronal circuits in SNr. This may be solved by multi-unit recording methods.

### Resource Availability

#### Lead Contact

Further information and requests for resources should be directed to and will be fulfilled by the Lead Contact, Kazutaka Maeda (kaz.maeda.86@gmail.com).

#### Materials Availability

Materials and the information used for the experiments are available upon reasonable request.

#### Data and Code Availability

Original data have been deposited to Mendeley Data: [https://doi.org/10.17632/g2shdsbtgt.1].

## Methods

All methods can be found in the accompanying Transparent Methods supplemental file.

## Supplemental Information

Document S1. Transparent Methods, Figures S1–S7, and Tables S1 and S3

Table S2. Statistic Values, Related to Figures 1–6
