# Supplementary material for: Primate Amygdalo-Nigral Pathway for Boosting Oculomotor Action in Motivating Situations
Source: iScience. 2020 May 23;23(6):101194. doi: 10.1016/j.isci.2020.101194 (PMC7281789; doi:10.1016/j.isci.2020.101194)
Supplement: Document S1. Transparent Methods, Figures S1–S7, and Tables S1 and S3 [file mmc1.pdf]

iScience, Volume *23*

Supplemental Information

Primate Amygdalo-Nigral Pathway for Boosting

Oculomotor Action in Motivating Situations

Kazutaka Maeda, Ken-ichi Inoue, Jun Kunimatsu, Masahiko Takada, and Okihide Hikosaka

Supplementary Figures

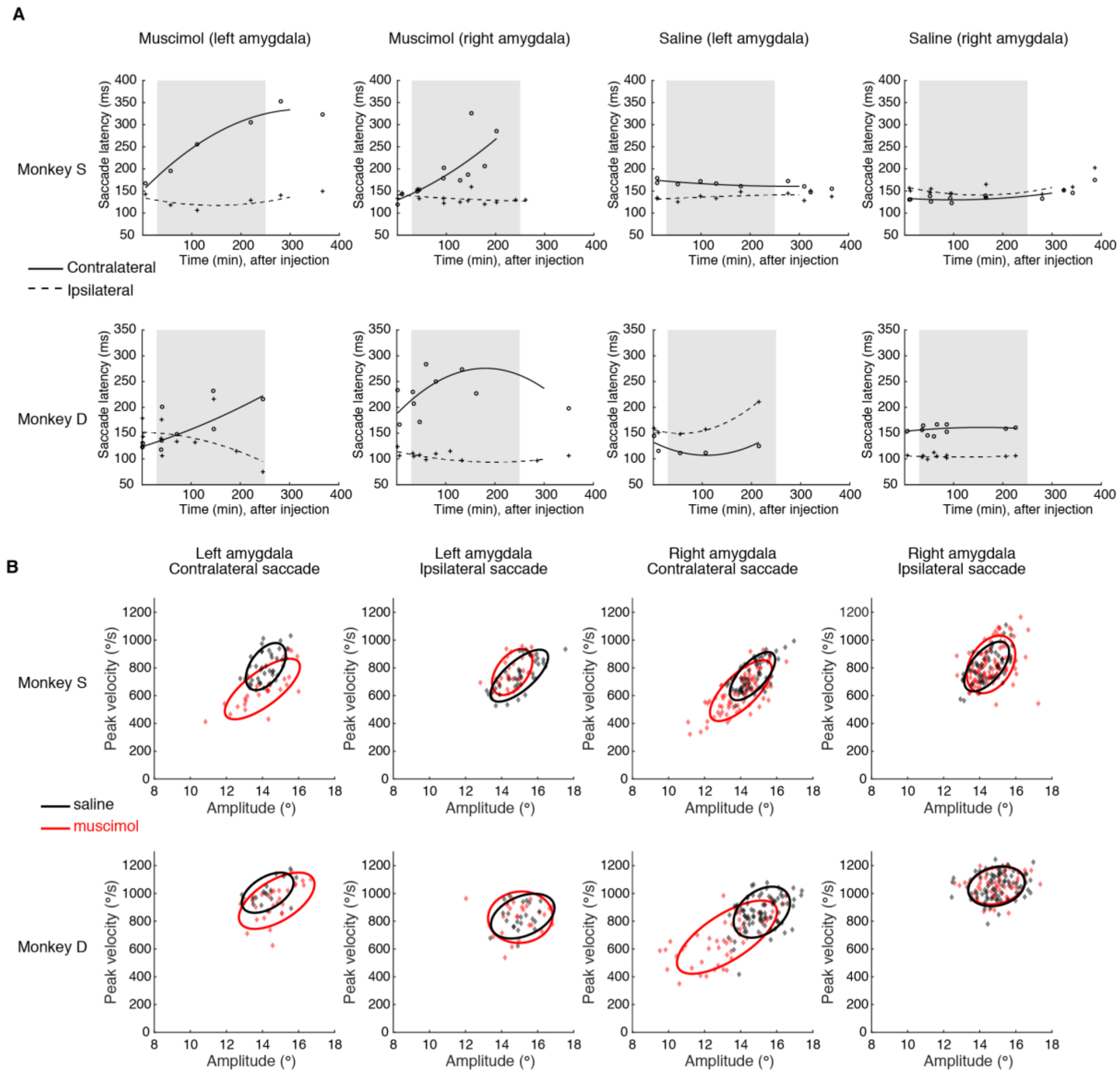

Figure S1. Change in saccade latency, amplitude, and peak velocity following amygdala inactivation (related to Figure 1).

A, Original data of Figure 1D (monkey S) and data from another subject (monkey D) **B**, Original data of Figure 1F (monkey S) and data from another subject (monkey D).

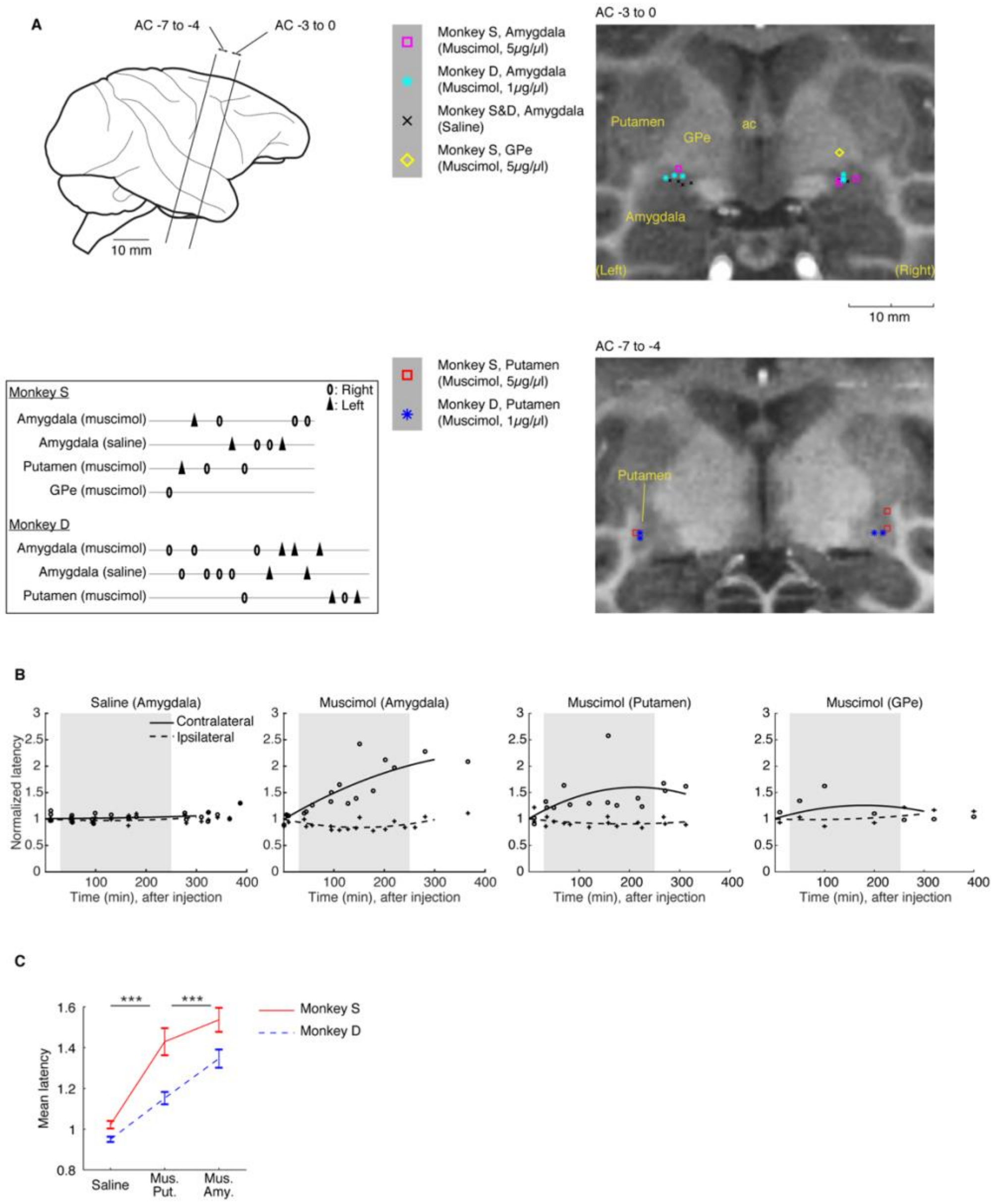

Figure S2. Change in saccade latency following inactivation of the amygdala and surrounding areas (related to Figure 1).

A, Estimated injection sites in the amygdala, GPe, and the putamen. The sites are shown in merged coronal MR images spanning 0-3 mm posterior (top) and 4-7 mm posterior (bottom) to the anterior commissure. ac, anterior commissure. The order of the injections in each monkey is shown in the left-bottom panel (from left to right). **B**, Changes in saccade latency after injection of saline into the amygdala, muscimol into the amygdala, the putamen, and GPe (Data from monkey S). Same format as Figure 1d. **C**, Mean saccade latencies for three conditions (saline injection into the amygdala, muscimol injection into the putamen, and muscimol injection into the amygdala). The individual data point shows the mean latency in each monkey and each condition (red solid line: monkey S, blue dash line: monkey D). Error bar shows SEM. Asterisk (***) indicates statistically significant contrasts at $P < 0.001$ for nested data from both monkeys (one-way ANOVA, post hoc: Fisher's LSD).

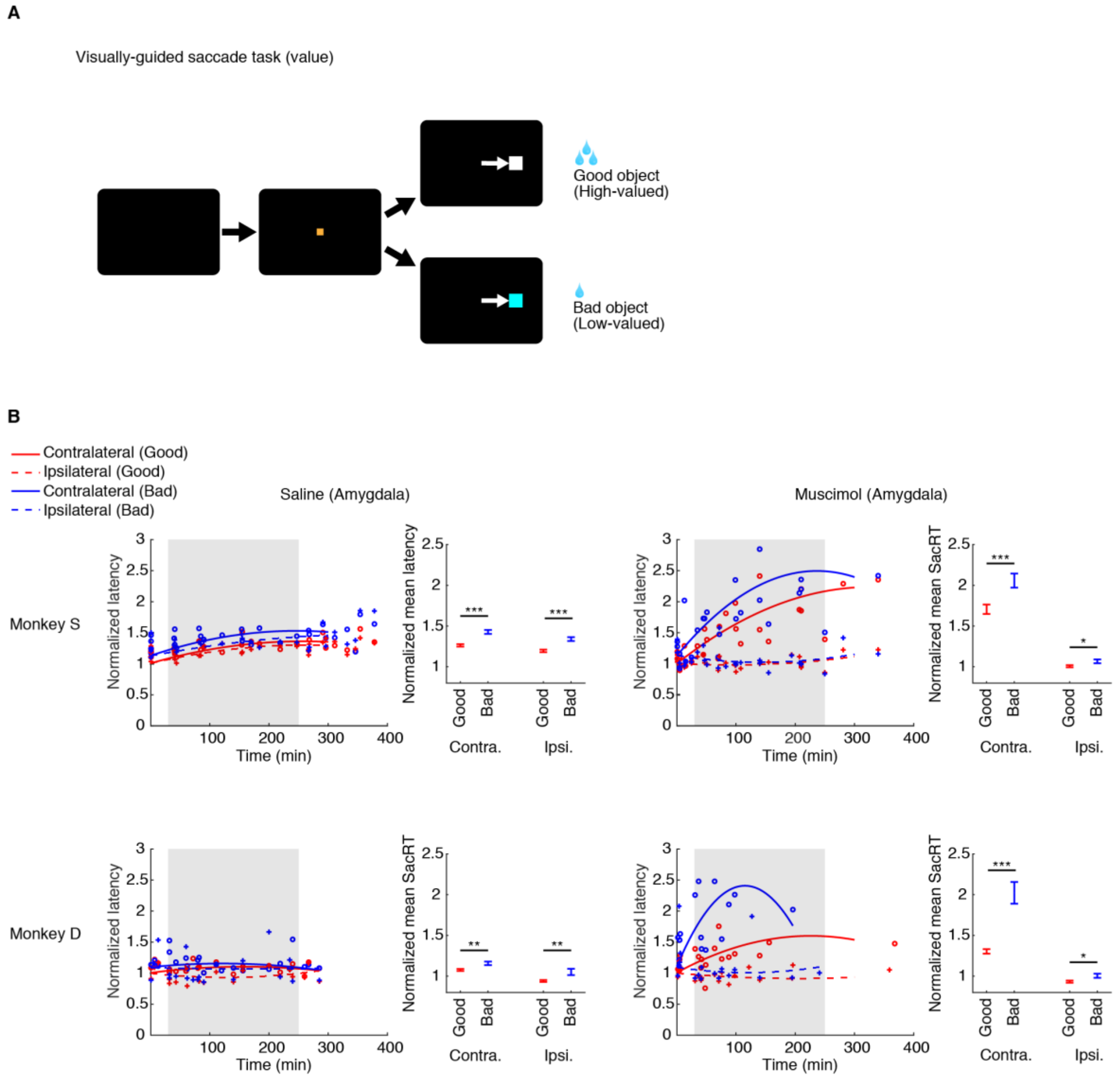

Figure S3. Change in saccade latency to objects of different values following amygdala inactivation (related to Figure 1).
A, Visually-guided saccade task, with target values differing across trials. The white dot (good object) was associated with high reward amount, and the cyan dot (bad object) was associated with low reward amount. **B**, Change in saccade latency by inactivation of the amygdala. Same format as Figure 1D. Asterisks (*), (**), and (***) indicate statistically significant contrasts at $P < 0.05$, $P < 0.01$, and $P < 0.001$ (two-sample t-test).

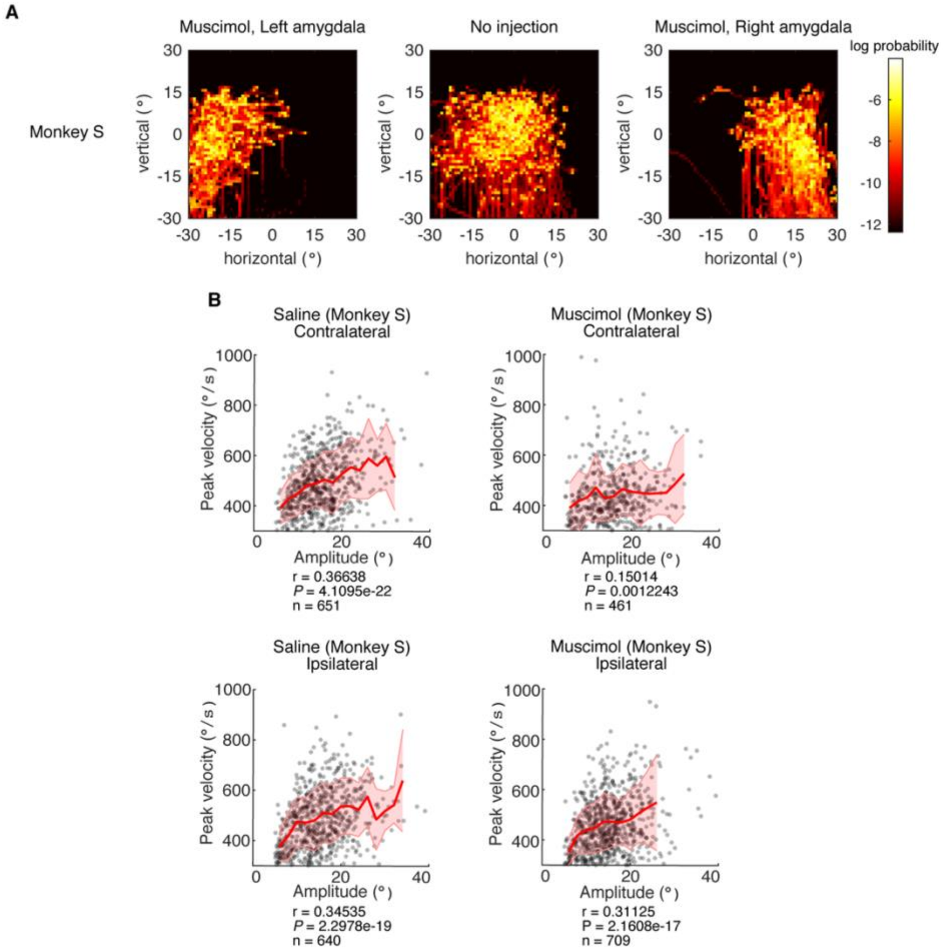

Figure S4. Changes in free viewing following amygdala inactivation (related to Figure 3).
A-B, Data from another subject (monkey S, same format as in Figure 3B and E).

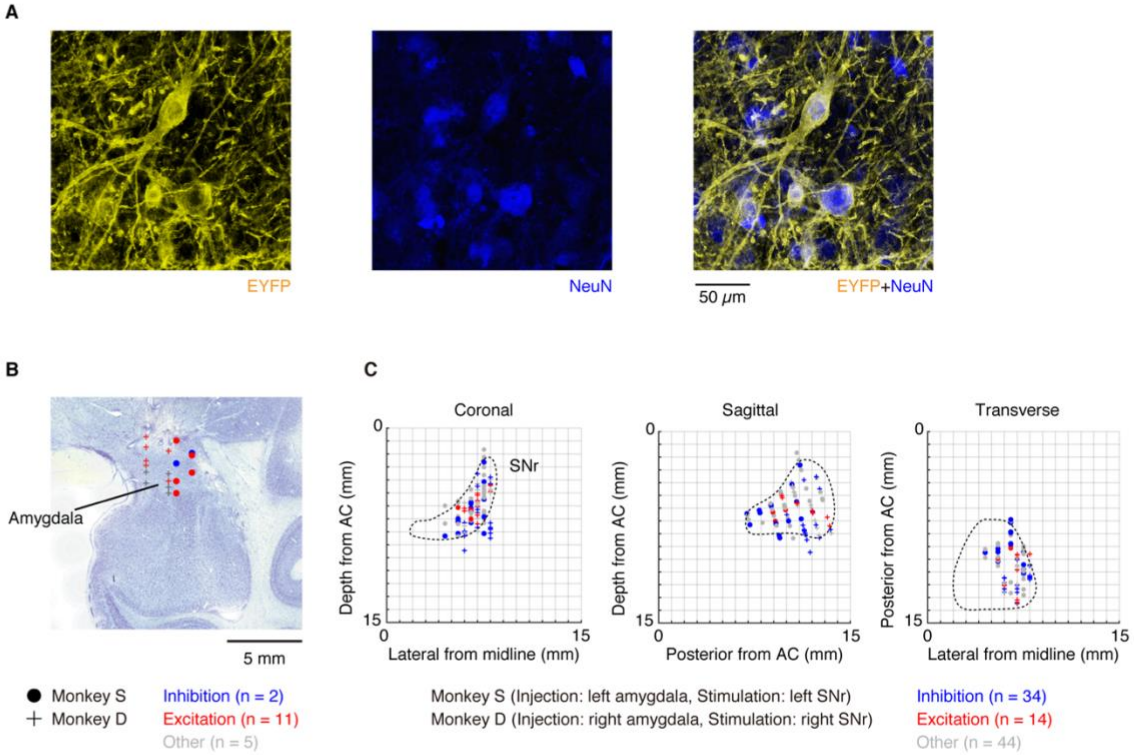

Figure S5. Optogenetic stimulation sites in the amygdala and SNr (related to Figure 4 and Figure 5).

A, Enhanced AAV2-CMV-ChR2-EYFP infection in the amygdala are shown in yellow (left). NeuN-expressing amygdala neurons are shown in blue (middle). Both are shown in the right panel. **B**, Stimulation sites in the amygdala. **C**, Estimated stimulation sites in the SNr.

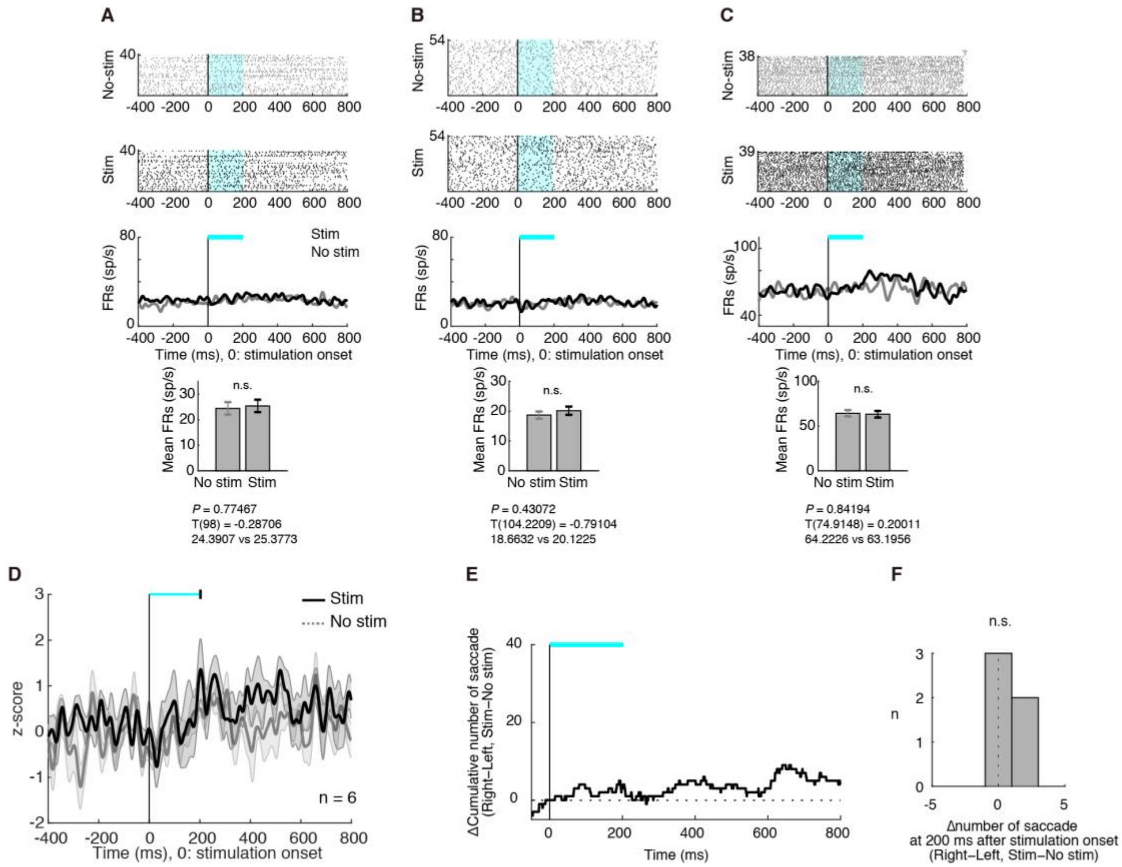

Figure S6. Control data for optogenetic stimulation (related to Figure 4 and Figure 5).
A, Optical stimulation to an example amygdala neuron in another subject (monkey T) without viral expressions. **B**, Optical stimulation of an example amygdala neuron of the right (contralateral) side in monkey S. **C**, Optical stimulation of an example SNr neuron of the right (contralateral) side in monkey S. **D**, Population activity by stimulation in monkey T. Stimulation did not lead to any clear modulation (two sample t-test, $T(7.2962) = -0.44275$, $P = 0.67078$). The error bars show SEM. **E**, Directional bias of saccades after right (contralateral) amygdala optical stimulation (n = 5) in monkey S. Same format as Figure 4C. **F**, Bias in the number of saccades during the stimulation of the right (contralateral) hemisphere (one-sample t-test, $T(4) = 1.5$, $P = 0.104$). Same format as Figure 4D.

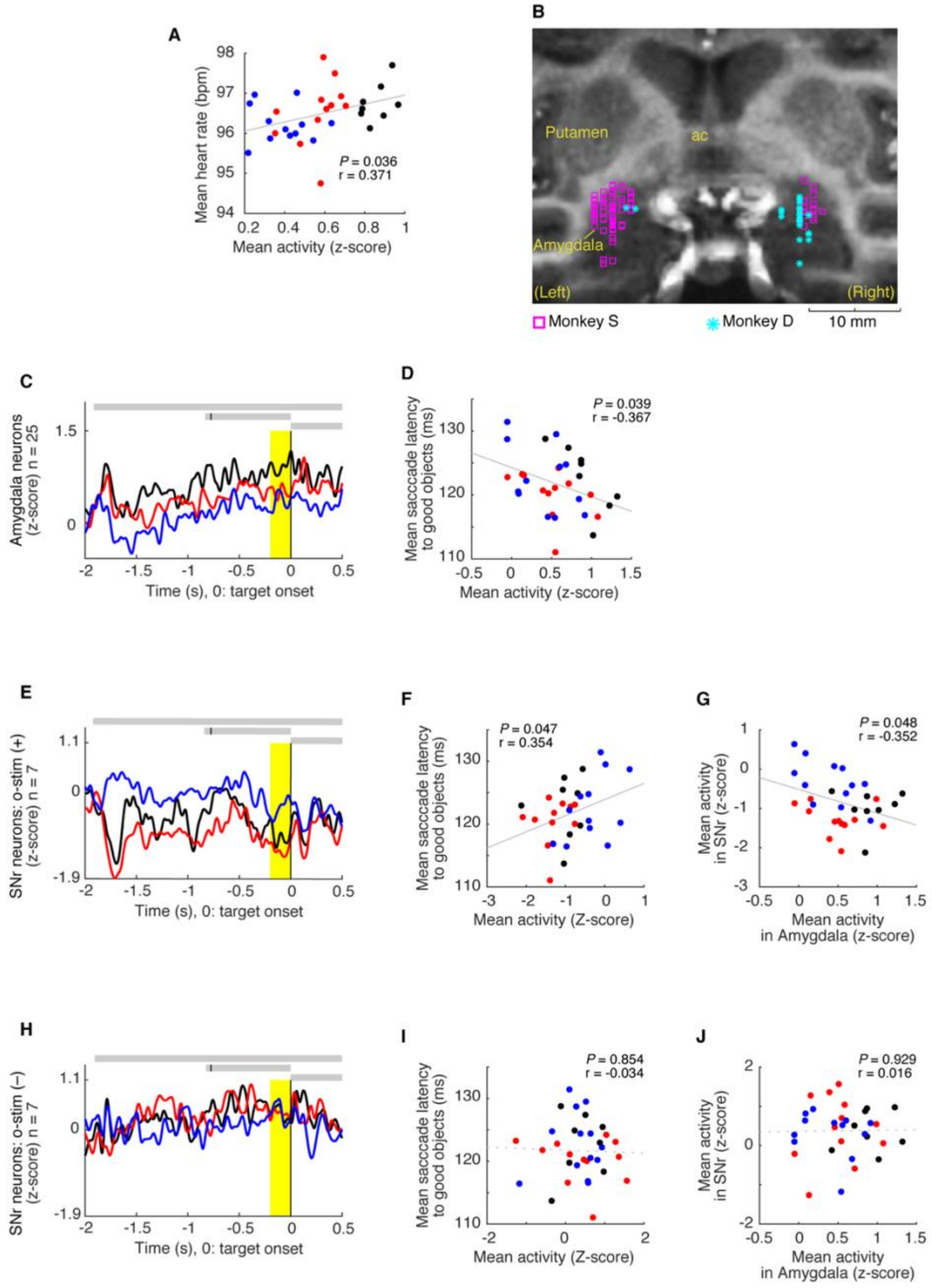

Figure S7. Recording of amygdala and SNr neurons (related to Figure 6).
A, Relation between the mean activity (abscissa) and mean heart rate (ordinate) for individual scenes (Pearson's correlation) in monkey S. **B**, Estimated recording positions. The sites are shown in merged coronal MR images spanning 0-3 mm posterior to the anterior commissure (n = 84 in monkey S, n = 25 in monkey D). ac, anterior commissure. **C**-**J**, Data from monkey D (same format as in Figure 6**B**, **D**, **E**, **G**, **H**, **I**, **K**, and **L**). **C-D**, Population activity of amygdala neurons. **E-G**, Population activity of SNr neurons that were optogenetically manipulated cells. **H-J**, Population activity of SNr neurons that were not optogenetically manipulated cells.

Supplementary Tables

Table S1 (related to Figure 1)

Detail of muscimol injection experiments for the amygdala (task VS: visually-guided saccade, task FV: free-viewing task, Mus.: muscimol). AC: anterior commissure. The positions listed below are chamber coordinates, which were tilted 15 degrees anteriorly relative to the stereotaxic reference frame (millimeter).

Monkey	Concn., µg/µl	Volume (µl)	Injection side	Posterior from AC (mm)	Lateral from midline (mm)	Depth from AC (mm)	Tasks
D	Mus. 1µg/µl	1	Left	2	9	5.9	VS, FV
D	Mus. 1µg/µl	1	Left	3	11	6.1	VS, FV
D	Mus. 1µg/µl	1	Left	1	10	5.8	VS, FV
D	Mus. 1µg/µl	1	Right	2	10	6.2	VS, FV
D	Mus. 1µg/µl	1	Right	2	10	5.7	VS, FV
D	Mus. 1µg/µl	1	Right	1	10	6.4	VS, FV
D	Saline	1	Right	2	10	6.3	VS, FV
D	Saline	1	Right	0	10	6.5	VS
D	Saline	1	Right	0	10	6.3	VS, FV
D	Saline	1	Right	2	10	6.5	VS, FV
D	Saline	1	Left	1	8	6.7	VS, FV
D	Saline	1	Left	0	9	6.9	VS, FV
S	Mus. 5µg/µl	1	Left	1	9.5	5	VS, FV
S	Mus. 5µg/µl	1	Right	3	11.5	6.2	FV
S	Mus. 5µg/µl	1	Right	1	9.5	6.4	VS, FV
S	Mus. 5µg/µl	1	Right	1	9.5	6.7	VS, FV
S	Saline	1	Left	1	9.5	6.5	VS, FV
S	Saline	1	Right	1	9.5	6.2	VS, FV
S	Saline	1	Right	2	10.5	6.5	VS, FV
S	Saline	1	Left	2	7.5	6.4	VS, FV

Table S2 (related to Figures 1-6)
Statistic values. (the file was provided separately as an Excel table)

Table S3 (related to Figure 4)
Injection sites of the viral vector for optogenetic experiments. AC: anterior commissure. The positions listed below are chamber coordinates, which were tilted 15 degrees anteriorly relative to the stereotaxic reference frame (millimeter).

Monkey	Volume (μl)	Injection side	Posterior from AC (mm)	Lateral from midline (mm)	Depth from AC (mm)
D	2	Right	1	10	7.1
D	2	Right	2	9	7.1
S	3	Left	2	9.5	6.5
S	2	Left	1	10.5	6.5
S	2	Left	3	10.5	7

TRANSPARENT METHODS

Subjects and surgery

We used two rhesus monkeys (Macaca mulatta) (monkey S: 8.5 kg, 7y old, male, monkey D: 5.0 kg, 13y old, female). All animal care and experimental procedures were approved by the National Eye Institute Animal Care and Use Committee and complied with the Public Health Service Policy on the Humane Care and Use of Laboratory Animals. Both animals underwent surgery under general anesthesia during which a head holder and a recording chamber were implanted on the head. Based on a stereotaxic atlas (Saleem and Logothetis, 2007), we implanted a rectangular chamber targeting the amygdala. The chamber was tilted anteriorly by 15 degrees in both monkeys. After confirming the position of recording chamber using MRI (4.7 T, Bruker), a craniotomy was performed during a second surgery.

Neuronal recording

Single-unit recording was performed using glass-coated electrodes (Alpha-Omega). The electrode was inserted into the brain through a stainless-steel guide tube and advanced by an oil-driven micromanipulator (MO-97A, Narishige). The recording sites were determined by using a grid system that allowed recordings at 1-mm intervals in x- and y-directions, orthogonal to the guide tube. The input from the electrode was amplified (A-M Systems) with a band-pass filter (0.2–10 kHz; BAK). Neuronal spikes were isolated online using custom software implementing a voltage and time window discriminator (Blip). We identified amygdala and the surrounding areas (striatum, GPe, and SNr) based on MRI images and characteristic firing patterns in neurophysiological recordings. The MR images were obtained in sections parallel to the recording chamber, which was visualized with gadolinium that filled grid holes and the space outside the grid and inside the chamber. As the electrode was advanced, the characteristic activity of GPe neurons (high-frequency tonic firing around 30-100 Hz, and occasional pauses) faded (Hong and Hikosaka, 2008). Then, after the electrode passed through a quiet white matter region, spikes of amygdala neurons (heterogeneous firing patterns, relatively consistent firing with no pause) grew larger. The striatum includes the following characteristic activities. Medium spiny neurons were identified by their low baseline activity (1-10 Hz) and broad action potentials (Kunimatsu et al., 2019). Tonically active neurons exhibited a characteristic tonic firing pattern and wider action potentials. Fast-spiking interneurons exhibited high baseline activity (around 30-80 Hz) and a sharp spike shape. SNr neurons exhibited consistent and high baseline activity (Yasuda et al., 2012).

Muscimol injections

Head position was stabilized throughout the experimental session by means of a chronically implanted head post. After the electrophysiological recording of amygdala neurons in both monkeys, we performed inactivation experiments to test for a causal relationship between amygdala neuronal activity and eye movements. We also inactivated surrounding areas of the amygdala (GPe and Striatum). To accurately inactivate the brain structure, we used an electrode assembly (injectorode) consisting of an epoxy-coated tungsten microelectrode (FHC) for unit recording and a polyimide tube (MicroLumen) for drug delivery. After the precise identification of the recording areas by unit recording, we injected the $GABA_A$ receptor agonist muscimol (Sigma, MO, USA) into the amygdala (8.8 nmol [1µg] or 44 nmol [5 µg] in a 1 µl volume) or other areas (44 nmol [5 µg] in a 1 µl volume) in one hemisphere at the speed of 0.2 ml/min by a 10 µl Hamilton syringe and manual infusion pump (Stoelting, IL, USA). Because the effect of muscimol on the amygdala lasted for several hours, inactivation sessions were limited to twice per week. The order of these injections was alternated. The minimum actual time between inactivation sessions was one day. Soon after the injection was completed (within 5 min), the animal was invited to start several tasks and repeat performance after periodic rest breaks every 20-60 min for 3-5 hr. In this experiment, we used a visually-guided saccade task and a free-viewing task to analyze the effects of inactivation on eye-movements. To show injection sits and recording sites on an image (Figure 1 and Figure S7), we first identified the anterior commissure and the amygdala on coronal sections of MR images in each monkey. Then, image co-registration was performed for these images based on the landmarks and superimposed by overlay function in Photoshop (Adobe Inc).

Viral injections and optogenetics

After the muscimol injection experiments were completed, we injected an adeno-associated virus type 2 vector (AAV2-CMV-ChR2-EYFP: 9.0 x 10^{12} genome copy/ml) into the amygdala area of one hemisphere in both monkeys (monkey S: left amygdala, monkey D: right amygdala). The vector was successfully used

in the macaque brain in a previous study (Inoue et al., 2015). Two penetrations in monkey D and three penetrations in monkey S were made into one side of amygdala at least 1.41 mm apart from each other. For each penetration, 2 µl (for monkey D) and 2 or 3 µl (for monkey S) of the vector were introduced at a rate of 0.4 µl/min for the first 0.2 µl, followed by 0.08 µl/min for the remainder of the injection controlled by a 10 µl Hamilton syringe and motorized infusion pump (Harvard Apparatus, Holliston, MA, USA).
For optical stimulation and electrophysiological recording, we used optrodes consisting of an epoxy-coated tungsten microelectrode (FHC) for unit recording and an optic fiber (200 µm diameter, Doric Lenses) for optical stimulation using laser light. The light sources were 473nm DPSS blue light lasers with a maximum power of up to 100mW (Opto Engine LLC). We left the laser on continuously during the experiment and placed a mechanical switcher (Luminos Industries Ltd) in the light path to turn the laser on and off. We measured the light intensity at the tip of the optrode before penetration of the brain using an optical power meter (1916-C, Newport Corporation) coupled with a 818-SL/DB photo detector. The light intensities were 30-50 mW/mm_2 for amygdala neurons and 300-500 mW/mm_2 for other projection sites (GPe, Striatum, and SNr). The maximum light intensity in each site was used for the controls as for the amygdala and SNr optical stimulations (50 mW/mm_2 and 500 mW/mm_2, respectively). Stimulation and non-stimulation periods were pseudo-randomly interleaved 100 times while the monkey was freely viewing during multiple visual stimulation regimes presented at random, including pictures, a blank screen, and the videos shown in the free-viewing task.

Histology

After completing all experiments, one subject (monkey D) was deeply anesthetized with an overdose of sodium pentobarbital (390 mg/ml) and perfused transcardially with saline followed by 4% paraformaldehyde. The head was fixed to the stereotaxic frame, and the brain was cut into blocks in the coronal plane including midbrain region. The block was post-fixed overnight at 4 C°, and then cryoprotected for one week in increasing gradients of glycerol solution (10 to 20% glyceorol in PBS) before being frozen. The frozen block was cut into 50 µm sections using a microtome. Slices taken at 250 µm-intervals were used for identifying EYFP signals and the adjacent slices were used for Nissl staining. We captured the fluorescent images of the labeled neurons and Nissl staining images using two different microscopes (Leica SP8 and Keyence BZ-X700). EYFP signals were excited by 514 nm wavelength laser and detected by HyD sensor (range from 519 nm to 640 nm wavelength). Autofluorescence signals were excited by same wavelength laser and detected by HyD sensor (range from 646 nm to 794 nm wavelength). We adjusted the contrast and brightness of each color channel using Photoshop (Adobe) to enhance the ability to identify fluorescently labeled neurons.

Immunofluorescence histochemistry

For enhancing the EYFP signals, we used rabbit anti-GFP antibody (G10362; Thermo Fisher) and goat anti-rabbit IgG antibody conjugated with Alexa fluor 488 (A-11034; Thermo Fisher). The sections were preincubated for 30 min in 0.3 % hydrogen peroxide in 0.1 M PBS (pH 7.4) to block endogenous peroxidase, followed by three rinses through 0.1 M PBS, and then 1 hour in blocking solution containing 5% normal goat serum in 0.1 M PBS. The sections were incubated for 18 hours at room temperature in blocking solution containing 2.5% normal goat serum and 0.1% TX-100 with rabbit anti-GFP antibody (1:2000). After three rinses with PBS, the sections were incubated for 2 hours at room temperature with goat anti-rabbit IgG antibody conjugated with Alexa fluor 488 (1:200). To visualize colocalization of ChR2-EYFP and neurons accurately, we have done double-staining by using chicken anti-NeuN antibody (1:500; ABN91, Millipore) and goat anti-chicken IgY (IgG) conjugated with Alexa fluor 594 (1: 200, A-11042; Thermo Fisher) in addition to the above method. We captured the fluorescent images of the labeled neurons and axons using the microscopes (SP8, Leica), range from 493 nm to 601 nm wavelength for the Alexa fluor 488 signals and range from 607 nm to 746 nm wavelength for autofluorescence signals or range from 596 nm to 760 nm wavelength for Alexa fluor 594. We adjusted the contrast and brightness of each color channel using Photoshop (Adobe) to enhance the ability to differentiate fluorescently labeled neurons.

Experimental control

All behavioral tasks were controlled by a custom neural-recording and behavior-controlling system (Blip; available at http://www.robilis.com/blip/). The monkey sat in a primate chair facing a fronto-parallel screen in a sound-attenuated and electrically shielded room. Visual stimuli were rear-projected onto a screen by

a digital light processing projector (PJD8353s, ViewSonic). Eye position was sampled at 1 kHz using a video-based eye tracker (EyeLink 1000 Plus, SR Research).

Foraging task

We previously devised a foraging task in which the monkeys viewed many scenes presented in randomized order across different trials and made saccades to good objects to receive rewards (Maeda et al., 2018). The detailed descriptions of methods should be referred to the paper. After a blank screen (inter trial interval 4 – 8 sec.), a scene appeared suddenly, and the subject was allowed to view the scene freely for 1,080 ms (FV period). Then, a fixation point (FP) appeared at the center. If the subject held its gaze on the FP for 780 ms (FX period), an object appeared at the same time as the FP disappeared. The object appearance was random and unpredictable in two ways: (1) sequence: two or three objects contained in the scene appeared randomly in sequence and (2) position: the objects appeared randomly at one of eight positions (eccentricity: 15 deg; angle: in steps of 45 deg from straight up). Each scene contained at least two objects with different features. Objects #1 and #2 were responsive to the subject's choice (i.e., sustained gaze, when the monkey made a saccade to it within 1,000 ms and kept fixating for >400 ms). Saccades to object #1 gave the subject a reward (called "good" object), whereas saccades to object #2 gave no reward (called "bad" object). Thus, the subject's goal was to make saccades to object #1 but not object #2. Such objects were present in all scenes, although they were visually different. Some scenes also contained the third object (object #3, "robber"). After the robber object appeared, it remained in place (irrespective of the subject's eye movements) until either the good or the bad object appeared. If the bad object appeared, the robber object did nothing, and the subject simply needed to make no action before FP reappeared. If the good object appeared, the robber was programmed to jump across the screen and intercept the object with timing designed to race against the monkey's saccade to the good object. On trials in which the robber's jump preceded the monkey's response, the robber "stole" the reward that would otherwise follow a correct saccade to the good object. Stolen trials occurred if the subject's saccade was delayed (reaction time: >105 – 135 ms for monkey D, >95 – 125 ms for monkey SO; the threshold was random across trials between these numbers). Otherwise, the subject obtained the reward. If the subject failed (i.e., the robber beat the saccade), the same trial was repeated in the same sequence until a successful trial occurred (i.e., the saccade beat the robber), but these repeated trials were not included in behavioral and neuronal analysis. To encourage the subject, the criterion reaction time was incremented by 10 ms after each failure. Based on the effects of these objects, the scenes were classified into three contexts. In the Safe and Rich (S/R) context, the good object was associated with a big reward (0.3 mL) and the robber object never appeared. In the Safe and Poor (S/P) context, the good object was associated with a small reward (0.1 mL) and the robber object never appeared. In the Dangerous and Rich (D/R) context, the good object was associated with a big reward (0.3 mL) and the robber object might appear but only did so on around 30 % of the trials randomly.

Visually-guided saccade task

Trials began with the appearance of a central fixation point. After the monkey fixated for 770 ms the fixation point disappeared, and a saccade target (2 x 2 degree) was presented at one of six positions randomly. The monkey was required to make a saccade and maintain fixation for 400 ms within the target window (10 x 10 degree) to obtain a water reward (0.1 mL). In Figure S3, two different reward amounts were used (0.08 and 0.15 mL for low- and high-valued object, respectively).

Free-viewing task

The monkey freely watched a movie without any rewards. The movie consisted of several video clips and lasted 5 minutes (30 frames/sec), mostly showing macaque monkeys engaged in natural behavior. We used the same movie across experiments. The video clips were assembled from commercially available documentaries and wildlife footage and previously used in another study (McMahon et al., 2015). The movie was presented within a rectangular frame of 46° wide and 34° high by custom-written MATLAB functions.

Data analysis for neuronal responses

To find visually responsive amygdala neurons, we let the monkey continue to perform the foraging task and checked responses to scene images and object images. We examined any neuron systematically if it responded to at least one scene image using the foraging task. Some SNr neurons were examined in the

foraging task while the neurons were modulated by optogenetic stimulation. We compared the responses of amygdala or SNr neurons to different contexts as well as individual scenes. To assess effects on neuronal activity that were purely attributable to scene images in the foraging task, we set a test window immediately before the saccade to the first object during FX period (200 ms before the object onset). We calculated the mean firing rate evoked by each scene for each neuron, which was converted to normalized rates: (FRi − FRb) / SD, (FRi: mean firing rate during the test window, FRb: baseline firing rate, SD: standard deviation of mean firing rates for all scenes). We then averaged normalized rates of all scene-responsive neurons for each context of scenes and for each scene. To examine the significance of context discrimination we used a one-way ANOVA (three groups [D/R, S/R, S/P], post hoc: Tukey–Kramer).
To investigate neuronal responses to the optical stimulation, we compared neuronal activities in a 200 ms test window after the stimulation onset with a baseline window (0–500 ms before the stimulation onset) for each neuron. Neuronal responses were considered significant whenever the corresponding statistics was associated with a P value that was less than 0.05 (t-test, inhibition or excitation; Figure 4 and 5).

Data analysis for saccade
In the visually-guided saccade task and foraging task, saccade latency was measured as the time between the offset of the fixation point (simultaneous with the onset of the object) and the onset of the saccade to the object. To determine effects on saccade latency purely attributable to scene images or contexts in the foraging task, the latency was measured only for saccades to the first good object in each trial. First, saccades were detected when the peak velocity of the eye trace exceeded 300 degrees/s. Saccade onset time was defined as the time point preceding the detected saccade at which the velocity exceeded 30 degrees/s. In the free-viewing task, we analyzed saccades with amplitudes ranging from 5 to 40 degrees. To calculate the gaze shift index, the gaze probability on the ipsilateral side in the injection condition was divided by the probability in the condition without injection. In the optogenetic stimulation experiments, differences in the cumulative number of saccades between stimulation and control conditions were plotted, including all sessions in each monkey. Saccadic eye movements to right and left directions were applied to positive and negative numbers, respectively. Positive values indicate more saccadic eye movements to right side compared with control data, and vice versa.

Data analysis for heart rate
Heart rate was measured by using a pulse sensor (World Famous Electronics llc) at the ear. The signals that were more than three times the median absolute deviations away from the median were removed as outliers in each trial. Then, a vector with the local peaks of the signals was returned to calculate inter-pulse intervals and converted to beats per minute. Outlier inter-pulse intervals were detected and removed when the duration was more than 450 ms. To determine effects on heart rate purely attributable to scene images or contexts before saccade initiation in the foraging task, we only used a most recent inter-pulse interval before first object onset in the FX period in each trial.

Supplemental References

Hong, S., and Hikosaka, O. (2008). The Globus Pallidus Sends Reward-Related Signals to the Lateral Habenula. Neuron *60*, 720-729.

Inoue, K.-i., Takada, M., and Matsumoto, M. (2015). Neuronal and behavioural modulations by pathway-selective optogenetic stimulation of the primate oculomotor system. In Nat Commun, pp. 8378.

Kunimatsu, J., Maeda, K., and Hikosaka, O. (2019). The Caudal Part of Putamen Represents the Historical Object Value Information. The Journal of neuroscience : the official journal of the Society for Neuroscience *39*, 1709-1719.

Maeda, K., Kunimatsu, J., and Hikosaka, O. (2018). Amygdala activity for the modulation of goal-directed behavior in emotional contexts. In PLoS Biol, pp. e2005339.

McMahon, D.B.T., Russ, B.E., Elnaiem, H.D., Kurnikova, A.I., and Leopold, D.A. (2015). Single-unit activity during natural vision: diversity, consistency, and spatial sensitivity among AF face patch neurons. In J Neurosci, pp. 5537-5548.

Yasuda, M., Yamamoto, S., and Hikosaka, O. (2012). Robust representation of stable object values in the oculomotor Basal Ganglia. The Journal of neuroscience : the official journal of the Society for Neuroscience *32*, 16917-16932.
